# From Exercise Dose to Exercise Architecture: Lifestyle-Congruent Exercise Models for Contemporary Adults

**DOI:** 10.3390/sports14070304

**Published:** 2026-07-17

**Authors:** Mario Muñoz-López, Edgar Simón Sancho-Haro, Aitor Zabaleta-Korta, Alexandra Martín Rodríguez, José Francisco López-Gil, José Francisco Tornero-Aguilera, Rodrigo Yánez-Sepúlveda

**Affiliations:** 1 Department of Sport Sciences, Faculty of Sport and Health Sciences, Fit Generation Research Institute, AD500 Andorra la Vella, Andorra; mario.mlopez@fitgeneration.es (M.M.-L.); aitorzabaletakorta@gmail.com (A.Z.-K.); jtornero@fitgeneration.es (J.F.T.-A.); 2 Department of Nutrition and Dietetics, Faculty of Sport and Health Sciences, Fit Generation Research Institute, AD500 Andorra la Vella, Andorra; 3 Faculty of Health Sciences, UNIE Universidad, Av. de Monforte de Lemos, 28, Fuencarral-El Pardo, 28029 Madrid, Spain; 4 School of Medicine, Universidad Espiritu Santo, Samborondon 0901952, Ecuador; josefranciscolopezgil@gmail.com; 5 Vicerrectoría de Investigación y Postgrado, Universidad de Los Lagos, Osorno 5290000, Chile; 6 Faculty of Education and Social Sciences, Universidad Andrés Bello, Viña del Mar 2200055, Chile; rodrigo.yanez.s@unab.cl

**Keywords:** lifestyle-congruent exercise, exercise architecture, exercise friction, exercise snacks, VILPA, movement breaks, sedentary behavior, physical activity adherence

## Abstract

Background: Contemporary adults face fragmented schedules, sedentary digital work, cognitive load, logistical barriers, and unstable routines that can make conventional session-based exercise difficult to sustain. Exercise snacks, vigorous intermittent lifestyle physical activity (VILPA), movement breaks, low-volume high-intensity interval training (HIIT), workplace exercise, and digitally mediated physical activity may reflect a broader redesign of exercise for real-world lifestyles. Objective: To develop a conceptual framework for lifestyle-congruent exercise models: approaches that intentionally modify dose, timing, setting, delivery, autonomy, or routine integration to reduce lifestyle-related friction while preserving a plausible physiological or behavioral stimulus. Methods: This evidence-based narrative review purposively identified sources through targeted searches, citation tracking, and manual verification across exercise science, sedentary behavior, workplace health, behavioral medicine, and digital health. Sources were selected for conceptual relevance, model-family representation, evidence-type diversity, and contribution to framework development, and then charted by model family, population, context, exercise architecture, delivery, outcomes, and implementation features. No systematic protocol, formal risk-of-bias assessment, or certainty grading were applied. Results: Thirty-one sources informed the framework. Five design logics were identified: dose compression, temporal distribution, routine embedding, sedentary substitution, and delivery mediation. These logics show that brief or time-efficient models are not interchangeable; they differ in intentionality, context, behavioral demand, safety considerations, implementation logic, and dominant friction addressed. Conclusions: Lifestyle-congruent exercise models are complementary strategies, not replacements for structured aerobic and muscle-strengthening exercise. Their value lies in matching exercise architecture to lifestyle-related friction while supporting progression toward guideline-consistent activity. Future research should test safety, equity, scalability, and long-term maintenance across diverse populations.

## 1. Introduction

Physical activity guidelines have progressively moved away from a narrow view of exercise as a fixed, session-based behavior and now recognize that health-enhancing movement can be accumulated through different combinations of frequency, intensity, duration, and muscle-strengthening activity [1,2]. The 2020 World Health Organization (WHO) guidelines recommend that adults accumulate 150–300 min of moderate-intensity aerobic physical activity, 75–150 min of vigorous-intensity activity, or an equivalent combination across the week, together with muscle-strengthening activities on two or more days per week [1]. Contemporary guidelines also emphasize that some physical activity is better than none and that sedentary time should be limited and, where possible, replaced with activity of any intensity [1,3]. This shift broadens the conceptual space for prescribing movement, but it does not by itself solve the practical problem of fitting exercise into contemporary adult life.

Despite this flexibility at the guideline level, many exercise prescriptions and fitness programs still implicitly rely on a high-friction model of participation: planned sessions, dedicated time blocks, stable routines, facility or equipment access, attentional bandwidth, and sustained motivation [1,2]. Such models remain valuable and should not be framed as obsolete, since guideline-consistent aerobic and muscle-strengthening activity remains the long-term reference standard [1,2]. However, they may fit poorly with how many adults now organize work, family responsibilities, and self-care, particularly in contexts shaped by sedentary routines, fragmented schedules, and reduced opportunities for uninterrupted activity [3].

Sedentary behavior research further challenges the view of exercise as only discrete leisure-time activity. Prolonged sitting is associated with adverse health outcomes, and interrupting it with brief light- or moderate-intensity bouts can improve acute cardiometabolic responses such as postprandial glucose and insulin [3,4,5]. This has driven interest in movement breaks and distributed activity that reorganize movement around the temporal, occupational, and environmental structure of daily life, rather than merely compressing exercise into shorter sessions.

The common explanation that adults are inactive because they “lack time” is therefore incomplete. Time scarcity is not merely a quantitative barrier measured in minutes, but part of a broader exercise-friction problem involving temporal fragmentation, transition costs, planning burden, cognitive switching, environmental constraints, and competing social roles. Time-efficient formats respond to this differently: low-volume HIIT compresses a planned session to improve cardiorespiratory fitness and cardiometabolic markers with lower volume, though tolerability, affect, and safety vary across populations [6,7,8], whereas exercise snacks redistribute brief bouts such as stair climbing across the day [9,10]. These models are conceptually important but should not be treated as interchangeable.

Vigorous intermittent lifestyle physical activity (VILPA) is a related but distinct development. Rather than prescribing formal exercise, VILPA refers to short bursts of vigorous activity embedded into daily living, such as fast stair climbing, brisk uphill walking or carrying loads. Wearable-based cohort evidence from UK Biobank has associated small daily amounts of VILPA with lower mortality among adults reporting no leisure-time exercise [11]. This evidence is associative and cannot exclude confounding by health status, functional capacity, or unmeasured lifestyle factors, so it should not be read as causal proof that brief vigorous movement can replace structured exercise; rather, it shows how device-based measurement reveals routine-embedded activity sitting between deliberate exercise and daily living.

Digital and wearable-mediated approaches add a further layer. Mobile apps, activity trackers, remote coaching, and wearable feedback can support prompts, self-monitoring, goal setting, and asynchronous delivery. Syntheses suggest that wearable trackers and eHealth or mHealth interventions can increase physical activity, although effects are modest, heterogeneous, and challenged by weak long-term engagement [12,13]. Digital mediation is therefore not inherently beneficial or a model of exercise in itself: its value depends on whether it reduces friction or adds barriers such as notification fatigue, privacy concerns, inequitable access, and low digital literacy.

**Figure 1:**
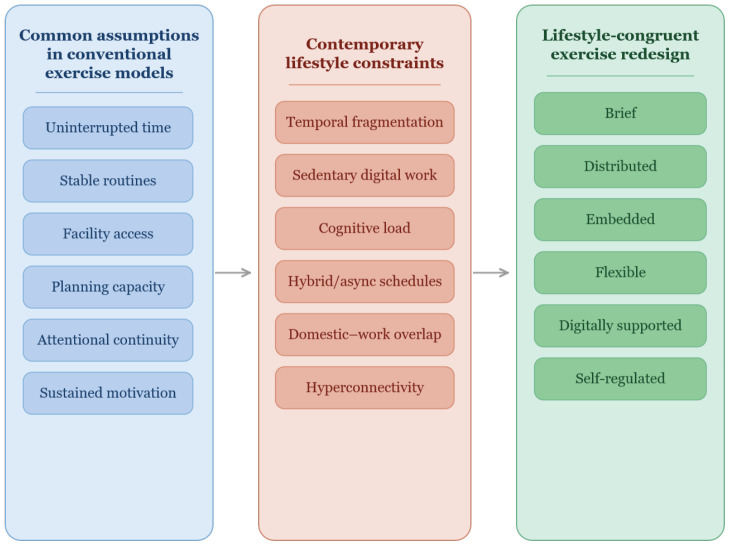
Conceptual rationale for lifestyle-congruent exercise models. Assumptions often embedded in conventional exercise models include uninterrupted time, stable routines, facility access, planning capacity, attentional continuity, and sustained motivation. Contemporary adult lifestyles may challenge these assumptions through temporal fragmentation, sedentary and digitally mediated work, cognitive load, hybrid or asynchronous schedules, domestic–work overlap, and hyperconnectivity. Lifestyle-congruent exercise models are proposed here as an author-derived umbrella concept for approaches that seek to reduce friction between exercise participation and daily life by using brief, distributed, embedded, flexible, digitally supported, or self-regulated formats. These models are intended to complement, rather than replace, structured exercise when conventional prescriptions have low contextual fit.

Taken together, exercise is being redesigned along several dimensions: duration, distribution, setting, intentionality, supervision, technology use, autonomy, and integration into routines. We use “lifestyle-congruent exercise models” as an author-derived organizing concept for approaches designed, prescribed, or adopted to intentionally reduce one or more forms of friction between movement and contemporary adult life while preserving a plausible physiological or behavioral stimulus. A model is not lifestyle-congruent merely because it is brief, digital, or convenient; it becomes so when its architecture improves the fit between movement and daily life without eliminating the exercise signal that gives it value (Figure 1).

This umbrella concept includes, but is not limited to, exercise snacks, physical activity snacks, VILPA, low-volume HIIT, minimal-dose resistance training, movement breaks, sedentary behavior interruptions, workplace and home-based exercise, app- or wearable-supported activity, and flexible or autoregulated formats. They are grouped not because they are uniformly brief or technology-supported, but because they modify one or more elements of exercise architecture to improve contextual fit. The concept is distinct from adjacent constructs: “incidental physical activity” is everyday non-exercise movement rather than a prescribed architecture with a physiological target; VILPA is a narrower vigorous subset of lifestyle-embedded movement in free-living conditions [11]; and “lifestyle-integrated exercise” (e.g., the LiFE program) embeds pre-specified balance and strength tasks into daily routines in older adults [14]. Lifestyle-congruent models are broader, spanning designed programs and opportunistic approaches, and are defined by the design logic of reducing the mismatch between exercise architecture and contemporary adult life.

An evidence-based narrative review with conceptual framework development is warranted because this literature is dispersed across exercise physiology, sedentary behavior, behavioral medicine, public health, workplace health, digital health, and fitness technology. Existing reviews often focus on individual model families, such as HIIT, sedentary behavior interruptions, wearables, or digital interventions, rather than examining how these approaches collectively reflect a broader reconfiguration of exercise for real-world adult lifestyles [4,8,10,12,13]. Critically, the absence of a shared design-logic language for these models limits cross-model comparison, hinders friction-sensitive prescription, and makes it difficult to design interventions that explicitly target the most relevant behavioral barriers in a given population. Therefore, the purpose of this article is not to exhaustively identify every primary study within each subfield or to estimate comparative effectiveness, but to integrate purposively selected and conceptually informative evidence sources to clarify model boundaries, develop a taxonomy, and propose a translational framework for lifestyle-congruent exercise models. Two clarifications are warranted at the outset: the taxonomy and the constructs proposed here (exercise architecture and exercise friction) are author-derived and not yet empirically validated, and should be read as a hypothesis-generating framework; and their novelty is integrative, recombining established prescription variables (FITT-VP) and behavior-change theory (COM-B, the Fogg model, self-determination theory) into a single context-sensitive design language rather than replacing them.

Accordingly, this review aims to describe how brief, distributed, time-efficient, flexible, digitally mediated, home-based, workplace-based, routine-embedded, and minimal-dose resistance models are conceptualized, delivered, and translated into practice. Specifically, it clarifies the design logics that distinguish these models, identifies the lifestyle-related friction each attempts to reduce, and proposes a practical framework for matching exercise architecture to adult life contexts. It does not assume that brief or digitally supported exercise should replace structured, guideline-consistent activity [1,2]; rather, it starts from the premise that different adults need different exercise architectures depending on the dominant friction in their lives. The central question is not whether contemporary models are superior to conventional ones, but how exercise can be redesigned as a lower-friction, context-sensitive complement to structured physical activity.

## 2. Methods

### 2.1. Review Design

This article was designed as an evidence-based narrative review with conceptual framework development, consistent with the narrative review type described by Grant and Booth [15]. This approach was selected because the aim was not to estimate pooled effectiveness, formally grade certainty of evidence, make comparative recommendations, or exhaustively identify every primary study within each subfield. Instead, the objective was to integrate purposively selected and conceptually informative evidence sources across several emerging exercise and physical activity model families to clarify model boundaries, develop a conceptual taxonomy, and propose a translational framework. This review did not follow the formal protocols of a systematic or scoping review, including Arksey and O’Malley or Levac frameworks, and was not reported according to PRISMA-ScR. It therefore adopts an explicitly purpose-driven narrative approach, justified by the early-stage and dispersed nature of the topic, where the primary need is conceptual clarification rather than exhaustive evidence synthesis or comparative effectiveness assessment. To increase transparency and reduce concerns about selection bias, the identification and selection process is reported in detail below, and the framework’s status as a purpose-driven, non-systematic synthesis is stated explicitly.

The review focused on models that appear to reduce the mismatch between exercise participation and contemporary adult life by modifying one or more elements of exercise architecture: dose, timing, distribution, setting, delivery mode, autonomy, intentionality, or integration into daily routines. The methodological emphasis was therefore placed on conceptual relevance, model differentiation, dominant friction addressed, and translational utility rather than on quantitative aggregation.

### 2.2. Conceptual Scope

The central concept was lifestyle-congruent exercise models, defined as exercise or physical activity approaches designed, prescribed, or adopted to intentionally reduce one or more forms of lifestyle-related friction while preserving a plausible physiological or behavioral stimulus.

The review considered five broad design logics:

1. Dose compression: models that reduce session duration, total exercise volume, or weekly training commitment while preserving a planned exercise or training structure, such as low-volume HIIT, time-efficient training, or minimal-dose resistance training.
2. Temporal distribution: models that divide physical activity into multiple short bouts across the day, such as exercise snacks or physical activity snacks.
3. Routine embedding: models that integrate movement into existing daily tasks or routines, such as vigorous intermittent lifestyle physical activity.
4. Sedentary substitution: models that interrupt or replace prolonged sitting with brief movement, such as movement breaks, active microbreaks, or sedentary behavior interruptions.
5. Delivery mediation: models in which the delivery context or support system is modified through workplace structures, apps, wearables, remote delivery, or hybrid formats.

These categories were used as organizing concepts rather than mutually exclusive classifications, because several models combine more than one design logic. Delivery mediation was treated as a support layer that can prompt, monitor, adapt, or deliver several lifestyle-congruent models, rather than as a specific exercise dose or bout structure.

### 2.3. Evidence Identification

Candidate evidence sources were identified through targeted literature searching, citation tracking, and manual verification of key records across the relevant model families. Searches were designed to locate conceptually informative sources for framework development rather than to provide an exhaustive inventory of all primary studies. Priority was given to reviews, meta-analyses, scoping reviews, umbrella reviews, framework papers, qualitative studies, and key empirical studies that contributed to model definition, conceptual differentiation, implementation interpretation, safety considerations, or translational relevance for lifestyle-congruent exercise models.

Search terms were organized around the following model families and concepts: “exercise snacks”, “physical activity snacks”, “activity snacks”, “vigorous intermittent lifestyle physical activity”, “VILPA”, “movement breaks”, “active breaks”, “sedentary breaks”, “active microbreaks”, “breaking up prolonged sitting”, “low-volume HIIT”, “brief HIIT”, “time-efficient exercise”, “minimal-dose exercise”, “low-volume resistance training”, “distributed physical activity”, “home-based exercise”, “workplace exercise”, “app-guided exercise”, “mobile health”, “mHealth”, “eHealth”, “wearable-guided exercise”, “activity trackers”, “remote exercise”, “hybrid exercise”, “flexible exercise”, “autoregulated exercise”, “exercise friction”, and “exercise architecture”. Concretely, targeted searches were run in PubMed/MEDLINE, Scopus, Web of Science, and Google Scholar, complemented by backward and forward citation tracking of key reviews and framework papers. Searches combined model-family terms (e.g., “exercise snacks”, “physical activity snacks”, “VILPA”, “low-volume HIIT”, “movement breaks”, “breaking up sitting”, “minimal-dose resistance training”, “workplace physical activity”, “mHealth/wearable physical activity”) with concept terms (“time-efficient”, “adherence”, “feasibility”, “implementation”, “sedentary behavior”). Records in English published up to 2025 were considered, prioritizing the most recent and highest-tier syntheses (umbrella reviews, meta-analyses, and systematic reviews) for each family, with primary studies and qualitative work added to illustrate mechanism, feasibility, or barriers.

Search records and supplementary citation tracking procedures were used to verify that each included source contributed to model definition, differentiation, implementation, or translational interpretation. Reference lists of relevant reviews and key papers were also examined to identify additional conceptually informative sources. Publication details, DOI information, and source relevance were checked manually before inclusion in the evidence map.

### 2.4. Eligibility Criteria

Sources were eligible if they addressed adults aged 18 years or older and contributed to the definition, evaluation, implementation, or conceptual differentiation of exercise or physical activity models that modify dose, timing, setting, delivery, autonomy, or routine integration to reduce lifestyle-related friction.

Eligible contexts included general adult populations, insufficiently active or sedentary adults, office-based, desk-based, digital or hybrid workers, older adults, and adults with cardiometabolic risk factors, provided that the model was potentially transferable to non-elite, real-world adult contexts and addressed a modifiable source of exercise friction.

Sources were excluded if they focused exclusively on children or adolescents, elite athletes, military or tactical populations, rehabilitation-only clinical settings, or traditional supervised exercise programs with no clear adaptation to dose, timing, context, flexibility, delivery mode, or routine integration. Sources addressing generic physical activity promotion were excluded when they did not describe a clearly identifiable model of exercise or physical activity redesign relevant to contemporary adult life.

### 2.5. Selection of Evidence Sources

Identified records were screened for conceptual relevance to the review aim. Sources were prioritized when they helped define a model family, differentiate one model from another, synthesize evidence on brief, distributed, embedded, time-efficient or digitally mediated exercise, examine feasibility, adherence, acceptability, implementation or safety, or clarify how exercise models can be adapted to contemporary lifestyle constraints.

Included sources were classified according to their role in the conceptual framework. Core sources directly informed the definition, taxonomy, or synthesis of a model family. Secondary sources addressed a relevant model but had a narrower population, outcome, or clinical focus. Peripheral sources, including narrative or conceptual papers, were used only when they contributed to framing, terminology, or translational interpretation, and were not treated as primary evidence sources in the narrative synthesis. Candidate sources were identified, read, and screened by the lead author using targeted searches, citation tracking, and manual verification. The final evidence set was refined through coauthor discussion according to conceptual relevance, representation of model families, diversity of evidence types, and conceptual saturation. This selection process was deliberate and purpose-driven rather than exhaustive, and the resulting evidence base should be interpreted accordingly.

The final evidence set comprised 31 sources retained because they contributed directly or indirectly to model definition, taxonomy development, implementation interpretation or conceptual framework development. The figure of 31 sources reflects a purposive, saturation-based rather than exhaustive logic: sources were added family by family until additional records reproduced the design logics and implementation themes already captured without introducing a new model family, a new design logic, or a materially different implementation or safety consideration. Conceptual saturation was therefore operationalized as the point at which further sources no longer altered the taxonomy or the friction-to-architecture mapping. This threshold is appropriate for framework development but, by design, does not capture the full primary-study evidence base for any single family; the resulting map should be read as conceptually representative rather than quantitatively complete.

### 2.6. Data Charting

A structured charting form was used to extract and organize information from each evidence source. Extracted items included author, year, DOI, study design, population or context, model family, model definition, exercise or physical activity dose, bout or session duration, frequency, intensity, delivery mode, technology use, comparator where applicable, outcomes assessed, adherence or feasibility findings, acceptability, safety or adverse events, equity or accessibility considerations, dominant lifestyle-related friction addressed, and relevance to the lifestyle-congruent exercise framework.

A second layer of conceptual coding was applied to identify the primary design logic of each model: dose compression, temporal distribution, routine embedding, sedentary substitution, or delivery mediation. Where sources addressed more than one logic, the dominant logic was coded for the main table, and secondary logics were considered in the narrative synthesis to avoid forcing overlapping models into mutually exclusive categories.

### 2.7. Synthesis

Findings were synthesized descriptively and conceptually. First, evidence sources were organized by model family, study design, population, and setting. Second, models were grouped according to their primary design logic. Third, outcomes and implementation-relevant features were summarized across model families, including physical activity behavior, sedentary time, cardiorespiratory fitness, cardiometabolic markers, vascular outcomes, cognition, pain, well-being, adherence, feasibility, acceptability, safety, and equity. Finally, each model family was interpreted according to the dominant lifestyle-related friction it appeared to address.

The final narrative synthesis generated a taxonomy of lifestyle-congruent exercise models and a translational decision matrix linking adult life contexts, dominant lifestyle-related friction, and candidate exercise architectures. Model families were interpreted according to their dominant design logic and the primary lifestyle-related friction they appeared to address, while secondary logics were retained for narrative interpretation where relevant. No meta-analysis, formal risk-of-bias assessment, or certainty-of-evidence grading was conducted, as the purpose of the review was conceptual framework development rather than quantitative effect estimation or comparative recommendation.

### 2.8. Reflexivity and Methodological Boundaries

Because this article is an evidence-based narrative review with conceptual framework development rather than a systematic or scoping review, the evidence base should not be interpreted as an exhaustive inventory of all studies within each model family. The purpose was to integrate purposively selected and conceptually informative sources to clarify model boundaries, identify design logics, examine the lifestyle-related friction addressed by each model family, and propose a practical framework for exercise prescription and implementation.

This methodological boundary is important. The review does not determine comparative effectiveness between model families, establish causal effects, or provide graded recommendations. Instead, it offers an organizing framework that can inform future systematic reviews, intervention development, implementation studies, and practice-oriented exercise prescription. Accordingly, the taxonomy, exercise-architecture construct, and exercise-friction construct are explicitly provisional and hypothesis-generating: they require empirical operationalization and validation (construct, content, and predictive) before being treated as established tools, and this article should be read as a structuring proposal rather than as validated evidence.

## 3. Evidence Map and Model Families

### 3.1. Overview of the Evidence Base

The conceptual framework was informed by 31 purposively selected evidence sources retained for main or secondary synthesis. These sources included scoping reviews, systematic reviews, systematic reviews with meta-analysis, meta-analyses, an umbrella review, qualitative evidence, randomized crossover trials, a pilot intervention study, and selected review papers that directly informed the conceptualization of lifestyle-congruent exercise models [10,16,17,18,19,20,21,22,23,24,25,26,27,28,29,30,31,32,33,34,35,36,37,38,39,40,41,42,43,44,45,46]. The evidence base was heterogeneous by design, reflecting the fact that lifestyle-congruent exercise is not yet an established research category but rather an author-derived umbrella framework linking several partially overlapping literatures.

The included evidence sources were organized into several model families nested within five broad design logics (Table 1): brief and intermittent activity models, including exercise snacks and physical activity snacks; lifestyle-embedded vigorous activity, represented by VILPA; sedentary substitution models, including sedentary behavior interruptions, movement breaks and active microbreaks; time-efficient structured training, including low-volume HIIT and minimal-dose resistance training; and delivery-mediated models, including workplace, mobile health, app-guided, and wearable-supported interventions [10,16,17,18,19,20,21,22,23,24,25,26,27,28,29,30,31,32,33,34,35,36,37,38,39,40,41,42,43,44,45,46]. These families were not mutually exclusive. For example, workplace interventions may include movement breaks, digital prompts, or sedentary interruptions; exercise snacks may overlap with accumulated physical activity; minimal-dose resistance training shares the dose-compression logic with low-volume HIIT but targets muscle-strengthening rather than cardiorespiratory fitness; and wearable-guided models may support either structured exercise or incidental movement.

The included sources were not treated as evidence of equivalent weight. Reviews and meta-analyses were used to map broader evidence clusters, qualitative studies were used to identify barriers and enablers, and selected primary studies were used when they illustrated translational feasibility or implementation outside tightly controlled settings. This approach allowed the synthesis to prioritize conceptual relevance, model differentiation, and implementation meaning rather than aggregate effectiveness. Crucially, the model families do not carry equivalent evidentiary weight, and this asymmetry is made explicit in Table 2. Low-volume HIIT and, to a lesser degree, sedentary-substitution and minimal-dose resistance models are supported by meta-analyses of controlled trials, whereas exercise snacks remain an emerging evidence base of small, short trials, and VILPA rests largely on associative, UK Biobank-dominated cohort data with no randomized evidence. Across families, the literature is further limited by heterogeneous protocols and outcomes, a predominance of acute or short-term endpoints, small samples, likely publication bias toward positive and feasible results, and sparse long-term adherence data; these constraints temper any translation into practice and are weighed throughout the synthesis rather than smoothed over.

### 3.2. Brief and Intermittent Activity Models

Brief and intermittent activity models were represented by evidence on exercise snacks and physical activity snacks [10,16,17,18,19]. These sources describe short bouts of physical activity performed across the day rather than as a single continuous exercise session. The defining feature of this family is not simply low duration, but the redistribution of exercise into smaller units that may be easier to insert into daily routines.

Jones et al. mapped exercise snacks and other forms of intermittent physical activity across epidemiological, experimental, and qualitative evidence, highlighting potential benefits but also substantial heterogeneity in definitions, protocols, and outcomes [10]. François et al. provided early experimental evidence that brief exercise bouts performed before or after meals attenuate postprandial glycaemia in adults with insulin resistance, contributing a metabolic rationale to the exercise snack concept [16]. Alexe et al. focused on exercise snacks as a strategy to interrupt sedentary behavior, reinforcing the conceptual overlap between brief exercise bouts and sedentary substitution [17]. Brown et al. synthesized evidence on multiple short bouts of aerobic physical activity among adults, reporting health-related benefits and adherence signals that support the feasibility of accumulated short-bout formats [19]. Du et al. added an implementation-relevant perspective by examining perceptions and experiences of exercise snacks among middle-aged and older adults, identifying acceptability, simplicity, and integration into daily life as relevant themes [18].

Taken together, this model family suggests that exercise can be reorganized from a continuous time block into smaller, repeatable bouts. Its main lifestyle-congruent function is to reduce the need for uninterrupted time, travel, equipment, and formal preparation. However, substantial heterogeneity persists in how bout duration, intensity, frequency, daily volume, and progression are defined and reported across studies. A key implication for later conceptual synthesis is that exercise snacks should be distinguished from both low-volume HIIT and movement breaks: they may all be brief, but they differ in intensity, intentionality, structure, and behavioral function.

### 3.3. Lifestyle-Embedded Vigorous Activity: VILPA

VILPA was represented by a framework/scoping paper and a qualitative focus-group study [20,21]. This model is conceptually distinctive because it shifts attention from prescribed exercise sessions to vigorous opportunities embedded in daily life. Examples include fast stair climbing, brisk uphill walking, carrying heavy loads, or other brief vigorous efforts that occur within routine activities.

Stamatakis et al. proposed VILPA as a potentially scalable approach for physically inactive adults, especially those who do not participate in structured leisure-time exercise [20]. The model is important because it occupies an intermediate space between formal exercise, incidental activity, and free-living movement patterns detected by wearable devices. Thøgersen-Ntoumani et al. examined barriers and enablers among physically inactive adults and found that VILPA may be more acceptable when it is brief, purposeful, convenient, and linked to existing routines, while barriers include discomfort, low confidence, physical limitations, and uncertainty about what counts as suitable vigorous activity [21].

Within the lifestyle-congruent framework, VILPA primarily represents routine embedding. Unlike low-volume HIIT, it does not require a planned training session. Unlike movement breaks, it is not necessarily designed to interrupt sitting. Its value lies in transforming ordinary tasks into opportunities for physiologically meaningful movement. However, translation requires caution: VILPA may need clear examples, graded progression, risk stratification, and environmental opportunity to be implemented safely and equitably. Neither source provides controlled efficacy evidence for VILPA: Stamatakis et al. [20] is a scoping and framework paper that draws on epidemiological data from population cohorts using accelerometry-detected vigorous intermittent activity, not from randomized interventions. Thøgersen-Ntoumani et al. [21] is a qualitative acceptability study. Within the lifestyle-congruent framework, VILPA is therefore included on the basis of design-logic plausibility, population reach, and conceptual differentiation, not as a model with an established causal efficacy evidence base.

### 3.4. Sedentary Substitution: Movement Breaks and Interruptions to Sitting

Sedentary substitution models were represented by evidence on breaks in sedentary behavior, active microbreaks, movement breaks, and interruptions to prolonged sitting [22,23,24,25,26,27,28,29]. This was one of the most developed clusters in the evidence map. Its defining feature is not the compression of exercise into shorter sessions, but the replacement or interruption of prolonged sitting with brief movement.

Chastin et al. provided early meta-analytic evidence linking breaks in sedentary behavior with cardiometabolic health markers [27]. Peddie et al. demonstrated in a randomized crossover trial that breaking prolonged sitting with regular light-intensity walking bouts significantly improved postprandial glucose, insulin, and triglyceride responses compared with uninterrupted sitting, providing direct experimental support for the sedentary substitution design logic [29]. Whipple et al. focused on adults at risk for type 2 diabetes and examined acute effects of sedentary breaks on vascular health [23]. Chueh et al. synthesized evidence on the cognitive effects of breaking up prolonged sitting, showing that the potential relevance of sedentary interruption extends beyond cardiometabolic outcomes [24]. Swartz et al. addressed community-dwelling adults aged 60 years or older, indicating relevance for older adult populations [22]. Zabatiero et al. contributed evidence from adults with overweight or obesity, although this source was treated as secondary because of its narrower population focus [28].

Workplace-oriented movement break evidence further connects sedentary substitution to contemporary occupational life. Radwan et al. reviewed active microbreaks among office workers and reported potential relevance for physical and mental well-being [26]. Shakerkavar et al. was retained as a secondary source because it focused specifically on neck pain relief in office settings, but it remained relevant to the use of active breaks within sedentary work environments [25].

Within the lifestyle-congruent framework, sedentary substitution models address a different problem from “lack of exercise time”. Their primary target is the accumulation of uninterrupted sitting, particularly in desk-based, screen-based, or hybrid work. These models may be especially useful when adults are not ready or able to perform structured exercise but can interrupt sitting with brief, low-friction movement.

### 3.5. Time-Efficient Structured Training: Low-Volume HIIT

Low-volume HIIT and time-efficient structured exercise were represented by six evidence sources [33,34,35,36,37,38]. This family differs from exercise snacks, VILPA, and movement breaks because it generally preserves the structure of a planned exercise session while reducing total duration or exercise volume. Its primary design logic is therefore dose compression.

Sultana et al. synthesized evidence on low-volume HIIT and found modest but significant effects on body composition and cardiorespiratory fitness [35]. Yin et al. examined whether low-volume HIIT is a time-efficient strategy to improve cardiometabolic health and body composition [36]. Sabag et al. reviewed low-volume HIIT for cardiometabolic health and positioned it as a potentially efficient exercise strategy [34]. Reyes Sánchez et al. addressed low-volume HIIT in adults with insulin resistance, further linking this model to cardiometabolic risk contexts [33]. Wen et al. contributed broader evidence on HIIT protocols and VO_2_max improvements in adults [38]. Reljic et al. was included as a translationally relevant pilot study because it tested low-volume HIIT in a community setting, helping bridge laboratory efficacy and real-world feasibility [37].

This model family has a comparatively strong physiological rationale, particularly for improving cardiorespiratory fitness and cardiometabolic markers, though the evidence base is heterogeneous (high I^2^ on several outcomes in meta-analyses) and most controlled studies have been conducted in laboratory settings with screened populations. The translation to community and real-world contexts is supported by a smaller body of evidence, and outcomes from these settings should be distinguished from laboratory efficacy findings. The lifestyle-congruent value of low-volume HIIT should not be assumed solely from reduced duration. A short high-intensity session may reduce clock time but increase perceived effort, affective demand, recovery needs, screening requirements or safety concerns. Therefore, low-volume HIIT is best understood as one specific branch of lifestyle-congruent exercise: useful for some adults, but not necessarily the lowest-friction option for all.

### 3.6. Delivery-Mediated Models: Workplace, Mobile Health and Wearables

Delivery-mediated models were represented by evidence on workplace interventions, mobile health, app-supported activity and wearable- or smartphone-supported interventions [30,31,32,39]. These models differ from the previous families because the defining feature is often not a specific exercise dose or bout structure, but the modification of the environment, support system or delivery channel through which activity is promoted.

Buckingham et al. reviewed mobile health interventions aimed at increasing physical activity and reducing sedentary behavior in workplace settings [30]. Dehghan Ghahfarokhi et al. synthesized evidence from RCTs using wearable and smartphone applications in adults with overweight or obesity, contributing to the digital self-monitoring and feedback dimension of the framework [31]. Rouyard et al. provided broader umbrella-review evidence on workplace interventions targeting sedentary behavior and physical activity [32]. Abdin et al. reviewed physical activity interventions in office-based workplace settings and their effects on well-being [39].

**Figure 2:**
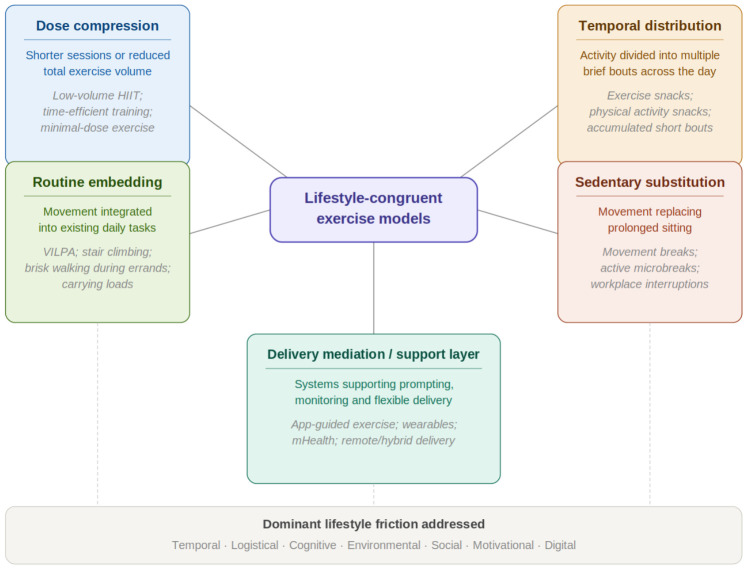
Taxonomy and design logics of lifestyle-congruent exercise models. Lifestyle-congruent exercise models can be organized into five design logics: dose compression, temporal distribution, routine embedding, sedentary substitution, and delivery mediation. The first four logics describe how movement is organized by modifying dose, timing, routine integration, or sedentary replacement. Delivery mediation differs slightly because it describes how movement is prompted, monitored, adapted, or delivered through digital, wearable, remote, or workplace systems. The taxonomy is author-derived and intended as a conceptual framework rather than a hierarchy of effectiveness.

Within the lifestyle-congruent framework (Figure 2), digital tools and workplace systems are best understood as delivery mediation rather than as independent exercise models. In this sense, delivery mediation functions as a transversal support layer: apps, wearables, and workplace programs can prompt movement breaks, guide short home-based sessions, monitor steps, support low-volume training, detect VILPA-like patterns, or provide feedback on sedentary time. Their practical value depends on whether they reduce friction by improving timing, feedback, accessibility, and self-regulation, or whether they add friction through complexity, notification fatigue, privacy concerns, or engagement decay.

### 3.7. Minimal-Dose Resistance Training

Physical activity guidelines recommend that adults perform muscle-strengthening activities involving all major muscle groups on two or more days per week [1,2], yet resistance training is often less adopted than aerobic activity in the general population. Common barriers include perceived time demands, uncertainty about technique, lack of equipment access, and limited exposure to programmed resistance exercise in community settings. Minimal-dose resistance training was therefore retained as a distinct model family within the dose-compression design logic, because it applies the question of time efficiency specifically to muscle-strengthening activity rather than to cardiorespiratory fitness.

Evidence on time-efficient resistance training suggests that session duration and weekly volume can be reduced by manipulating variables such as number of sets, exercise selection, rest periods, load, frequency, and proximity to muscular failure [40,41,42,43,44,45,46]. Low-volume protocols may be sufficient to initiate strength or hypertrophic adaptations, particularly in adults who are untrained, returning to exercise, or unlikely to adopt conventional resistance programs [41,42,45,46]. Within the lifestyle-congruent framework, the practical value of minimal-dose resistance training lies in making muscle-strengthening activity more accessible through brief whole-body routines, bodyweight exercises, resistance-band protocols, or minimalist free-weight sessions that require less time, equipment, or travel.

Several caveats are important. The evidence base is heterogeneous, and training responses vary by age, sex, baseline training status, exercise selection, intensity, proximity to failure, and progression strategy [40,41,42,43,44,45,46]. Minimal-dose resistance training should therefore be framed as a scalable entry point into muscle-strengthening activity, not as a permanent minimal prescription or a substitute for progressive overload. Technique guidance, perceived exertion monitoring, graded progression, and safety considerations remain essential, especially when resistance training is delivered at home, digitally, or without supervision.

### 3.8. Outcomes and Implementation-Relevant Features

Brief and intermittent activity models most commonly addressed feasibility, adherence, acceptability, cardiorespiratory fitness, and cardiometabolic markers [10,16,17,18,19], with this cluster drawing on systematic reviews and meta-analyses for broader estimates and a single controlled crossover trial for acute metabolic evidence [16]. VILPA sources focused exclusively on conceptual development, barriers, enablers, and behavioral acceptability [20,21]; no controlled efficacy trials were available, and health-outcome claims for this family rest on epidemiological evidence external to the included sources. Sedentary substitution models addressed sedentary time, cardiometabolic responses, vascular outcomes, cognition, pain, well-being, and workplace outcomes [22,23,24,25,26,27,28,29], supported by meta-analytic evidence [27,28] alongside individual RCT and systematic review data of variable scope. Low-volume HIIT sources focused mainly on cardiorespiratory fitness, body composition, and cardiometabolic health through systematic reviews with meta-analysis and narrative reviews [33,34,35,36,37,38]; one community-based pilot [37] contributed feasibility data only and should not be read as efficacy evidence. Minimal-dose resistance training sources drew on narrative reviews for time-efficient resistance training and health-related outcomes [40,43,44], meta-analyses for strength and hypertrophy outcomes [41,42,45], and one randomized controlled trial examining resistance-training volume in trained men [46]. Delivery-mediated models assessed physical activity behavior, sedentary behavior, digital engagement, self-monitoring, feasibility, and well-being across an umbrella review, a systematic review with meta-analysis of RCTs, and two systematic reviews [30,31,32,39].

Implementation-relevant features were inconsistently reported. Adherence, feasibility, and acceptability appeared frequently in brief-bout, VILPA, and digital sources, but definitions and measurement methods varied [10,17,18,19,20,21,30,31]. Safety and adverse events were reported unevenly, particularly for vigorous, high-intensity, or unsupervised formats. Equity and accessibility were also underdeveloped: several models assume access to safe stairs, flexible work breaks, home space, smartphones, wearables, digital literacy, or autonomy at work. These assumptions are important because lifestyle-congruent models may not be equally feasible across socioeconomic, occupational, functional, or environmental contexts.

Overall, and with the caveat that evidence quality ranges from meta-analyses of RCTs to single pilot studies and conceptual framework papers, the synthesis suggests that lifestyle-congruent exercise models should be evaluated not only by physiological outcomes, but also by behavioral and contextual fit. Measures such as perceived burden, autonomy, enjoyment, cognitive load, context compatibility, habit formation, long-term adherence, safety, equity, and dominant friction reduced are central to determining whether a model is truly congruent with adult life.

## 4. Conceptual Synthesis: From Exercise Dose to Lifestyle-Congruent Architecture

### 4.1. From “Time-Efficient Exercise” to “Low-Friction Exercise”

The evidence map suggests that contemporary exercise models are often described through the language of time efficiency, brevity, or convenience [6,7,8,9,10]. However, this terminology only partially captures the nature of the shift. The key transition is not simply from long to short exercise, but from high-friction to lower-friction exercise architectures. A model may be short but still difficult to implement if it requires high effort, specialized equipment, supervision, travel, recovery time, or strong motivational readiness [6,7,8,33,34,35,36,37,38]. Conversely, a slightly longer model may be more congruent with daily life if it is easy to initiate, embedded into routines, environmentally accessible, and cognitively simple.

Exercise friction can be understood as the cumulative behavioral cost of initiating, performing, and maintaining physical activity in a given life context [47,48]. This includes temporal, logistical, cognitive, environmental, motivational, social, and digital friction. Lifestyle-congruent exercise models attempt to reduce one or more of these frictions by changing the dose, timing, setting, delivery mode, autonomy, or integration logic of movement, while preserving a plausible physiological or behavioral stimulus.

The concept of exercise friction is consistent with established behavior-change frameworks but is used here as a practical design construct rather than as a separate behavioral theory. In Capability, Opportunity, Motivation–Behavior (COM-B) terms, friction may reduce capability, opportunity, or motivation to initiate and sustain movement [47]. In Fogg’s Behavior Model, friction can be understood as an ability-reducing force that prevents behavior even when motivation and prompts are present [48]. Self-determination theory further suggests that models preserving autonomy and competence may be more likely to support sustained participation [49]. In practical terms, exercise friction could be operationalized through measurable dimensions such as planning burden, transition cost, environmental access, cognitive demand, equipment requirements, supervision needs and compatibility with occupational, domestic, or caregiving roles. This framing helps distinguish models that are often grouped together. To move exercise friction from a descriptive concept toward an applicable design tool, these dimensions can be operationalized as a simple, provisional rating instrument (Table 3): each dimension is scored from 0 (low friction) to 2 (high friction), yielding a total “exercise-friction load” (0–16) whose dominant contributors indicate which lifestyle-congruent architecture is most likely to improve fit. This instrument is proposed for research use and requires psychometric validation before clinical application.

Low-volume HIIT primarily addresses temporal friction through dose compression, but may increase exertional burden, recovery demands, or safety considerations [33,34,35,36,37,38]. Exercise snacks address lack of uninterrupted time by distributing activity across the day, but require repeated initiation [10,17,18,19]. VILPA reduces formal planning demands by embedding vigorous movement into existing routines, but depends on environmental opportunity, physical confidence, and safe progression [20,21]. Movement breaks address sedentary accumulation by inserting activity into sitting time but require occupational permission and routine prompts [22,23,24,25,26,27,28]. Minimal-dose resistance training addresses time burden and facility-access friction through brief, low-set protocols requiring minimal equipment, but meaningful strength and hypertrophic adaptations depend substantially on proximity to muscular failure and progressive overload, which may require technical guidance, particularly for untrained populations [40,41,42,43,44,45,46]. Digital and workplace-mediated models may reduce access, feedback, or prompting barriers, but can also introduce digital, privacy, or organizational burdens [30,31,32,39].

### 4.2. Exercise Architecture as a Practical Design Construct

We propose exercise architecture as an author-derived organizing construct for describing how movement is configured in real life. Exercise architecture refers to the joint configuration of physical activity according to dose, bout duration, frequency, intensity, timing, setting, supervision, intentionality, delivery mode, transition demands, and degree of integration into daily routines. Two models may provide similar total exercise volume but differ substantially in feasibility, burden, safety requirements and contextual fit because their architectures differ. Although individual prescription variables such as intensity, frequency, duration, setting, and supervision are well established, their combined configuration as a contextual design parameter has not been formally operationalized. Future research should therefore examine how exercise architecture can be measured empirically using descriptors such as bout structure, required transition time, equipment, supervision, autonomy, prompting, safety procedures, and routine integration. In this sense, exercise architecture extends the familiar FITT-VP framework (frequency, intensity, time, type, volume, progression) by adding the contextual and behavioral dimensions—timing within the day, setting, supervision, intentionality, delivery mode, transition demands and routine integration—that determine whether a physiologically adequate dose is actually enacted. Operationally, a given prescription can be described as a profile across these descriptors and matched, via the exercise-friction score (Table 3), to the dominant friction it must overcome (Table 4), making the construct usable rather than purely descriptive.

This distinction is central for lifestyle-congruent exercise. A 15-min low-volume HIIT session, five 3-min exercise snacks, several active microbreaks during the workday, and three VILPA bouts accumulated during errands may all be brief. Yet they differ in how they are initiated, where they occur, how intentional they are, how much effort they require, what safety considerations they raise, and what type of behavioral support they need. Therefore, “brief exercise” should not be treated as a homogeneous category.

Exercise architecture also makes clear that physiological efficiency and behavioral feasibility are not the same. A model with a strong physiological stimulus may fail in practice if it is too aversive, difficult to schedule, unsafe for the target population or poorly matched to the person’s environment. Conversely, a model with a more modest physiological stimulus may still have public health value if it is widely adoptable, safe, scalable, and capable of moving inactive adults from no activity toward some activity. This is especially relevant for adults who are unlikely to adopt conventional structured exercise.

### 4.3. A Taxonomy of Lifestyle-Congruent Exercise Models

Based on the narrative synthesis, lifestyle-congruent exercise models can be organized into five design logics: dose compression, temporal distribution, routine embedding, sedentary substitution, and delivery mediation (see Figure 2). These logics are not mutually exclusive, but they clarify how different models modify exercise architecture to reduce specific forms of lifestyle-related friction. When a model fits more than one design logic, its primary classification is determined by the dominant design mechanism and the main source of friction it is intended to reduce: dose compression applies when the primary mechanism is reducing session duration or volume; temporal distribution applies when movement is redistributed into multiple bouts across the day; routine embedding applies when movement is integrated into existing daily tasks or routines; sedentary substitution applies when the main target is accumulated sitting; and delivery mediation applies when the defining feature is the support or delivery system rather than a specific exercise dose.

Dose compression refers to models that preserve a planned exercise or training structure while reducing session duration, weekly volume, or total time commitment. Low-volume HIIT and minimal-dose resistance training are the clearest examples within this logic, although they target different physiological domains: cardiorespiratory fitness and cardiometabolic health in the case of HIIT, and muscle strength, hypertrophy, or functional capacity in the case of resistance training [33,34,35,36,37,38,40,41,42,43,44,45,46]. These models primarily address temporal and logistical friction and may be useful for adults who can tolerate the required intensity, effort, or technical demands but lack time for longer sessions. However, reduced clock time should not be equated with universally lower burden, because intensity, recovery needs, affective responses, technique requirements, progression demands, and safety considerations may still create friction.

Temporal distribution refers to models that divide activity into multiple short bouts across the day. Exercise snacks and physical activity snacks fit this logic [10,16,17,18,19]. Their practical value lies in reducing the need for a single uninterrupted exercise window, although they may still require repeated initiation, simple prompts, and clear progression rules to avoid remaining at a trivial dose.

Routine embedding refers to models that integrate movement into existing daily tasks rather than requiring formal exercise planning. VILPA is the clearest example [20,21]. This logic may be particularly relevant for adults with low exercise identity or low willingness to engage in structured programs, because it reframes movement as part of daily living rather than as a separate exercise session. However, routine embedding depends on environmental opportunities, physical confidence, clear examples, and safe progression.

Sedentary substitution refers to models that insert movement into, or replace, prolonged sitting. Movement breaks, active microbreaks, and sedentary behavior interruptions fit this logic [22,23,24,25,26,27,28]. Their main target is not necessarily fitness improvement, but the reduction of uninterrupted sedentary exposure and creation of repeated low-friction opportunities for movement during sedentary contexts.

Delivery mediation refers to models in which apps, wearables, remote platforms, or workplace systems modify how physical activity is prompted, monitored, delivered, adapted, or supported [30,31,32,39]. This logic differs from the previous four because it does not define a specific exercise dose, bout structure, or movement pattern. Instead, it functions as a support layer that can be combined with several model families, including exercise snacks, movement breaks, low-volume HIIT, VILPA, or home-based routines.

### 4.4. Matching Exercise Architecture to Dominant Lifestyle-Related Friction

The practical implication of this taxonomy is that exercise prescription can be reframed as a matching problem. Instead of asking only which exercise dose is effective, practitioners and intervention designers can ask which exercise architecture best reduces the dominant lifestyle-related friction in a given context while preserving a meaningful physiological or behavioral stimulus, as shown in Table 4. This approach does not replace physiological reasoning; it complements it by adding behavioral and contextual fit.

For example, an adult with limited uninterrupted time but good tolerance for vigorous effort may be suited to low-volume HIIT or brief planned sessions. An office worker with prolonged sitting may benefit more from movement breaks, active microbreaks, or workplace activity prompts. A physically inactive adult with low interest in formal exercise may find VILPA or routine-embedded activity more acceptable. A digitally engaged adult may benefit from wearable-guided prompts, whereas another person may experience the same prompts as intrusive, distracting, or cognitively burdensome.

This interpretation also cautions against universal claims. No lifestyle-congruent model is inherently superior across adults or contexts. Each model solves some problems while potentially creating others. Low-volume HIIT reduces time but may increase exertional burden. Exercise snacks reduce the need for a continuous time block but require repeated initiation. VILPA reduces formal planning but depends on environmental opportunities and safe progression. Wearables provide feedback but may increase digital burden. The goal is therefore not to promote one model, but to select, combine, or progress models according to context.

The matrix should be interpreted as a translational aid rather than a clinical algorithm. It does not imply that brief or distributed models are sufficient for all adults, nor that they should replace guideline-based physical activity. Rather, it provides a practical way to identify feasible entry points, complementary strategies, and progression pathways when conventional exercise prescriptions have low contextual fit.

### 4.5. Avoiding Reductionism: Brief Does Not Mean Equivalent

A major risk in translating this literature is oversimplification. The message that “short exercise works” is appealing but incomplete. Short bouts vary widely in intensity, frequency, modality, intentionality, context, safety demands, and intended outcome. A brief vigorous bout, a low-intensity walking break, a stair-climbing snack, and a short HIIT session should not be treated as interchangeable simply because they are brief.

This matters for public health communication. Lifestyle-congruent models may help inactive adults initiate movement and may complement guideline-based activity, but they should not be framed as shortcuts that automatically replace aerobic and muscle-strengthening recommendations. The appropriate message is not that “a few minutes is enough,” but that smaller, better-integrated movement opportunities may reduce entry barriers, support progression, and help adults accumulate meaningful activity over time.

The same caution applies to digital interventions. Technology can support behavior change when it reduces decision burden, provides timely feedback, or enables flexible delivery. However, technology is not inherently lifestyle-congruent. It becomes congruent only when it reduces rather than increases behavioral, cognitive, or digital friction.

### 4.6. Implications for Future Intervention Design

Future interventions should be designed around the specific lifestyle-related friction they intend to reduce. If the target barrier is temporal fragmentation, the intervention should reduce the need for uninterrupted time. If the barrier is sedentary work, it should modify the structure of sitting. If the barrier is low exercise identity, it should minimize formal exercise demands and emphasize routine embedding. If the barrier is lack of feedback, digital support may be useful. If the barrier is cognitive overload, the model should be simple, low-decision, and minimally intrusive. In each case, the intervention should preserve a meaningful movement stimulus rather than only minimizing effort.

This approach implies that intervention reporting should move beyond standard exercise dose descriptors. Studies should report not only intensity, duration, and frequency, but also planning demand, setting, required equipment, transition costs, supervision, prompting, autonomy, progression, safety procedures, and compatibility with daily routines. These variables are central to understanding whether a model is truly lifestyle-congruent and reproducible outside controlled research conditions.

Future studies should also evaluate outcomes that capture behavioral fit and the specific friction targeted by the intervention. Alongside physiological markers, relevant outcomes include adherence, maintenance, enjoyment, perceived burden, cognitive load, planning effort, self-efficacy, autonomy, habit formation, contextual compatibility, digital engagement, adverse events, and equity. Without these measures, it is difficult to know whether a model is merely effective under study conditions, whether it actually reduces the intended lifestyle-related friction, or whether it is genuinely adaptable to adult life.

## 5. Translational Implications for Practice, Public Health, and Digital Fitness

### 5.1. Implications for Exercise Prescription

The lifestyle-congruent framework suggests that exercise prescription should move beyond asking whether a dose is effective under ideal conditions and also ask whether the proposed exercise architecture fits the person’s dominant lifestyle-related friction, daily context, and likely progression pathway. This does not replace established principles of exercise prescription; rather, it adds a contextual layer to them. Frequency, intensity, time, and type remain important, but they should be interpreted alongside timing, distribution, setting, autonomy, transition costs, cognitive burden, safety considerations, and compatibility with routines.

In clinical and community practice, lifestyle-congruent exercise selection should be guided by a brief friction assessment rather than generic prescription templates. General practitioners are well positioned to identify dominant exercise friction during routine consultations—asking not only “do you exercise?” but “what stops you?”—and to recommend friction-matched entry points before referral to structured programs. Physiotherapists and exercise physiologists can further individualize architecture selection based on functional capacity, movement competence, safety profile, and clinical context.

#### A Practical Implementation Pathway for Community and Clinical Settings

Translating this framework into practice requires specifying not only what is prescribed but where it is delivered, who delivers and supervises it, how participants are screened and educated, how progression and safety are managed, and how outcomes are evaluated. Table 5 outlines a pragmatic implementation pathway that assigns these components to concrete settings and professional roles, so that low-friction architecture is operationalized as a deliverable program rather than a description.

Economic implications are also relevant but should be framed cautiously. Minimal-dose and distributed models that require less equipment, travel, or supervision may reduce some participation costs and may improve access for adults without gym memberships, wearable devices, or flexible schedules. However, lower participation burden does not necessarily imply cost-effectiveness at the health-system or program level. Formal economic evaluations are needed to compare lifestyle-congruent models with supervised exercise programs, digital interventions, and conventional physical activity promotion strategies. This approach also supports a stepped model of exercise engagement. For adults who are currently inactive, the first goal may not be immediate achievement of full guideline targets, but initiation of sustainable movement through low-friction entry points that preserve a plausible movement stimulus.

Exercise snacks, short walking bouts, sit-to-stand breaks, stair use, or brief home-based resistance routines may help establish movement identity, confidence, and early habit formation before progressing toward higher volumes, added resistance, or more structured programs. Such progression should be explicit, because lifestyle-congruent models risk underdosing if they remain permanently minimal rather than serving as scalable entry points toward guideline-consistent aerobic and muscle-strengthening activity. Habit-formation research suggests that repeated behavior in stable contexts may reduce the motivational demand required to initiate activity over time, supporting the rationale for low-friction entry points that can be repeated and progressed [50].

### 5.2. Implications for Public Health Messaging

Public health communication often struggles to balance accuracy with simplicity. Messages such as “move more” or “sit less” are accessible but may be too vague to support behavior change, whereas detailed exercise prescriptions can appear unrealistic for adults with fragmented schedules, caregiving responsibilities, or sedentary digital work. Lifestyle-congruent models offer a middle ground by translating physical activity guidance into concrete, context-sensitive entry points that are easier to recognize, initiate, and progress.

A more practical public health message might emphasize that movement can be accumulated, distributed, and embedded into daily life, while still maintaining the importance of guideline-based aerobic and muscle-strengthening activity [1,3]. Rather than promoting a generic message that “any few minutes count”, communication could offer friction-matched examples: interrupt sitting during desk work, climb stairs briskly during routine transitions, accumulate short bouts across the day, add brief home-based resistance activity, or use prompts to reduce prolonged inactivity. This framing may provide a theoretically coherent communication strategy for adults who do not identify as exercisers and are unlikely to adopt conventional structured programs, because it translates recommendations into recognizable entry points rather than presenting them only as weekly targets. However, whether friction-matched exercise messages produce greater behavior change than generic physical activity promotion remains an empirical question; direct testing against standard exercise messaging should be considered a priority for future work in health communication and exercise behavior change.

Messaging must avoid overstatement. Brief or distributed models should not be presented as universal replacements for structured exercise, nor should VILPA, exercise snacks, or movement breaks be reduced to simplistic claims that “a few minutes is enough”. The more defensible message is that small, well-placed movement opportunities, when appropriately matched and progressed, can reduce entry barriers, complement structured exercise, and help inactive adults move toward more active, guideline-consistent patterns over time.

### 5.3. Implications for Workplaces

Workplaces are central to lifestyle-congruent exercise because they shape not only individual choices, but also the temporal, social, and environmental structure of much adult sedentary behavior. Desk-based and screen-based work can create long periods of uninterrupted sitting, high cognitive load, and limited perceived opportunities for movement. In this context, workplace interventions should not be viewed only as optional wellness add-ons aimed at individual motivation, but as modifications to work design and the organizational environment.

Movement breaks, active microbreaks, walking meetings, stair prompts, sit-stand transitions, and brief mobility or strengthening routines can be integrated into the workday with relatively low logistical cost when supported by workflow, managerial norms, and sufficient worker autonomy [22,23,24,25,26,27,28,32,39]. Their purpose is not necessarily to replace leisure-time exercise or guideline-based activity, but to reduce sedentary accumulation and create repeated opportunities for movement. Organizational support is therefore important: employees may be less willing to take movement breaks if workplace culture treats breaks as unproductive, if job demands provide little autonomy, or if movement opportunities are not compatible with workload.

Workplace implementation should also consider equity. Some workers have autonomy over breaks, access to stairs, flexible schedules, and safe environments; others do not. Office-based professionals may benefit from prompts and active breaks, whereas shift workers, service workers, or people in precarious employment may face different constraints. A lifestyle-congruent workplace strategy should therefore be adapted to job control, workload, physical environment, organizational norms, and worker input, rather than assuming that office-based solutions can be transferred unchanged across occupational groups.

### 5.4. Implications for Digital Fitness and Wearable Technologies

Digital tools may support lifestyle-congruent exercise when they reduce friction through timely prompts, simple feedback, remote guidance, self-monitoring, or flexible delivery. Wearables can make movement patterns visible, apps can deliver short routines, and remote platforms can reduce access barriers. However, their value is best understood as delivery mediation rather than as a standalone exercise model: they are useful insofar as they help implement brief, distributed, routine-embedded, or flexible movement with lower behavioral burden.

However, digital fitness should not equate more features with better support. Complex dashboards, excessive notifications, rigid streaks, social comparison, and constant self-monitoring may increase cognitive and digital friction. Privacy concerns, data burden, low digital literacy, and unequal access to smartphones or wearables may also reduce real-world congruence. A lifestyle-congruent digital tool should help the user act with less effort, not require continuous attention or additional technical work. This may involve simple prompts, context-aware suggestions, flexible goals, minimal data burden, transparent feedback, and options to reduce notification frequency.

Digital tools should also be designed for progression, safety, and responsible data use, particularly when they prompt vigorous, unsupervised, or stair-based activity. For example, a wearable prompt to take the stairs may be appropriate for some adults but not for others with mobility limitations, cardiometabolic risk, low fitness, or low confidence. Apps that prescribe brief high-intensity bouts should include pre-participation screening, contraindication prompts, graded progression, perceived exertion guidance, stop rules, and clear instructions; tools such as the Physical Activity Readiness Questionnaire for Everyone (PAR-Q+) may provide a practical basis for screening in digital delivery contexts [51]. Without these elements, digital delivery may reduce access barriers while scaling exposure faster than it scales safety. Because digital fitness applications may collect health and activity data, privacy, data minimization, and compliance with relevant data-protection frameworks should also be considered part of real-world congruence rather than an external technical issue.

### 5.5. Implications for Research and Implementation

The lifestyle-congruent framework highlights several priorities for future research. First, model reporting should be standardized. Studies should describe not only dose, frequency, and intensity, but also the dominant lifestyle-related friction targeted, bout structure, setting, prompting, equipment, supervision, progression, autonomy, transition demands, and routine integration. These features determine whether an intervention is genuinely compatible with daily life and whether its proposed mechanism of contextual fit can be replicated.

Second, behavioral fit and friction reduction should be measured directly. Alongside physiological and activity outcomes, studies should assess perceived burden, enjoyment, self-efficacy, cognitive load, planning effort, transition costs, habit formation, maintenance, digital engagement, contextual compatibility, and the extent to which the targeted lifestyle-related friction was actually reduced. These measures should be treated as core implementation and behavioral outcomes rather than optional additions, because physiological efficacy alone is insufficient if a model cannot be sustained outside research conditions.

Third, future studies should examine which adults benefit from which model, under which contextual conditions, and through which mechanism of friction reduction. Lifestyle-congruent exercise is unlikely to be one-size-fits-all. Comparative, pragmatic, or adaptive studies could test whether pre-specified friction profiles respond better to different architectures: dose-compressed sessions, distributed bouts, routine-embedded vigorous activity, sedentary substitution, or delivery mediation. Such research would move the field from generic feasibility claims toward context-sensitive exercise prescription and implementation.

Long-term adherence deserves particular emphasis, because for lifestyle-congruent models it is arguably the primary outcome: their rationale is sustained real-world participation rather than acute physiological potency, so a model that is efficacious in an 8–12-week trial but not maintained has limited public health value. Current evidence is weak on this point: most trials are short, digital and wearable interventions show well-documented engagement decay, and few studies report adherence beyond a few months or use a consistent adherence definition. Future work should treat sustained adherence, and its maintenance after support is withdrawn, as a primary endpoint, report it with standardized definitions and time horizons of at least 6–12 months, and analyze it against the specific friction the model was designed to reduce, distinguishing initiation from long-term maintenance.

Finally, implementation studies should address equity, safety, scalability, and maintenance. Models that depend on stairs, safe neighborhoods, smartphones, wearables, flexible work breaks, or home space may not be equally accessible. Safety reporting is especially important for vigorous, high-intensity, stair-based or unsupervised formats. Evaluative frameworks such as RE-AIM (Reach, Effectiveness, Adoption, Implementation, Maintenance) are well suited to this research agenda, assessing dimensions beyond efficacy that are central to understanding whether an intervention can scale and sustain across real-world settings [52]. The next phase of research should therefore ask not only whether lifestyle-congruent models can work, but for whom, under what contextual conditions, at what behavioral burden, with what safety profile, and whether they can be maintained safely, equitably, and sustainably across diverse adult populations.

## 6. Strengths and Limitations

This review has several strengths. First, it integrates evidence from research areas that are typically studied in isolation, including exercise snacks, physical activity snacks, VILPA, sedentary behavior interruption, movement breaks, low-volume HIIT, minimal-dose resistance training, workplace interventions, and digital physical activity support. Second, it proposes an author-derived conceptual taxonomy that classifies models according to their dominant design logic (i.e., dose compression, temporal distribution, routine embedding, sedentary substitution, and delivery mediation) rather than grouping them only by duration, intensity, or technology use. Third, it translates this taxonomy into a practical decision matrix linking adult life contexts, dominant lifestyle-related friction, candidate exercise architectures, and progression pathways, which may improve relevance for exercise prescription, public health messaging, workplace health, and digital fitness design.

Several limitations should also be acknowledged. This was an evidence-based narrative review with conceptual framework development rather than a systematic or scoping review; therefore, the evidence base was purposively selected and should not be interpreted as exhaustive. No systematic search protocol, PRISMA-ScR reporting, formal risk-of-bias assessment, or certainty-of-evidence grading was applied. The corpus was heterogeneous and included reviews, meta-analyses, qualitative evidence, an umbrella review, and selected empirical studies, which limits direct comparison between model families and prevents inference about relative effectiveness. Some model families, particularly VILPA, exercise snacks and minimal-dose resistance training, remain unevenly developed and require more long-term, adequately powered, and contextually diverse studies.

Additional limitations concern publication and reporting bias, external validity, and conceptual maturity. Studies reporting positive, feasible, or acceptable outcomes may be more likely to appear in the evidence sources consulted, potentially underrepresenting evidence of null effects, poor adherence, implementation failure, or adverse events. A substantial proportion of the evidence base comes from high-income settings, including the United Kingdom, Australia, North America, and Western Europe; VILPA evidence is strongly influenced by UK Biobank, and many workplace or digital studies assume stable employment, internet access, wearable ownership, or occupational autonomy. The generalizability of the framework to low- and middle-income contexts, informal employment, limited digital infrastructure, or constrained physical environments is therefore uncertain. Finally, the proposed concept and taxonomy of lifestyle-congruent exercise are author-derived and provisional; they should be refined through empirical testing, stakeholder input, implementation research, and periodic updating as new evidence and delivery formats emerge.

## 7. Conclusions

This evidence-based narrative review with conceptual framework development examined exercise and physical activity models designed or used to reduce the mismatch between movement and contemporary adult life. The purposively selected evidence sources suggest that exercise is being reconfigured beyond the conventional session-based model toward a broader repertoire of dose-compressed, temporally distributed, routine-embedded, sedentary-substitution, and delivery-mediated formats.

The proposed concept of lifestyle-congruent exercise models provides a framework for organizing this transition. These models differ in design logic: some compress exercise dose, others distribute activity across the day, embed vigorous movement into routines, substitute sedentary time with movement, or use digital and workplace systems to support flexible delivery. Their common feature is that they attempt to reduce one or more forms of lifestyle-related friction while preserving a plausible physiological or behavioral stimulus. It should be emphasized that this framework is a hypothesis-generating proposal: the taxonomy and the exercise-architecture and exercise-friction constructs are author-derived and await empirical validation, and the differing strength of evidence across model families (strongest for low-volume HIIT, emerging for exercise snacks, and largely associative for VILPA) means the models should not be presented to practitioners or the public as carrying equivalent support.

Lifestyle-congruent models should be framed as complementary strategies, not as replacements for structured exercise or shortcuts around physical activity guidelines. Their practical value lies in matching exercise architecture to dominant lifestyle-related friction, identifying feasible entry points, and supporting progression toward more active, guideline-consistent aerobic and muscle-strengthening patterns. Future work should empirically test whether such models can support sustained, equitable, safe, and scalable physical activity participation across diverse adult populations and settings.

## Figures and Tables

**Table 1:** Evidence sources informing the conceptual framework.

Ref.	First Author, Year	Evidence Type	Primary Model Family	Population/Context	Role in Conceptual Framework	Main Limitation(s) of the Evidence
[10]	Jones, 2024	Scoping review	Exercise snacks	Adults and older adults	Core	Scoping review; maps breadth without grading effect sizes or risk of bias.
[16]	François, 2014	Randomized crossover trial (RCT)	Exercise snacks/glycaemia	Adults with insulin resistance	Core	Small acute crossover; short-term postprandial outcome in an insulin-resistant subgroup.
[17]	Alexe, 2025	Systematic review	Exercise snacks	Adults; mixed health status	Core	Heterogeneous protocols; few long-term or hard-outcome data.
[18]	Du, 2024	Systematic review and meta-synthesis	Exercise snacks	Middle-aged and older adults	Core	Qualitative meta-synthesis; captures perceptions, not effectiveness.
[19]	Brown, 2024	Systematic review	Physical activity snacks	Adults	Core	Small, short-bout-based studies; limited adherence follow-up.
[20]	Stamatakis, 2020	Scoping review/framework paper	VILPA	Adults; inactive adults	Core	Conceptual framework/scoping paper; no effectiveness estimate.
[21]	Thøgersen-Ntoumani, 2023	Qualitative focus-group study	VILPA	Physically inactive adults	Core	Qualitative; small purposive sample; limited generalizability.
[22]	Swartz, 2024	Systematic review	Sedentary behavior interruption	Community-dwelling adults ≥60 years	Core	Predominantly acute/short-term outcomes; older adult focus; heterogeneity.
[23]	Whipple, 2021	Systematic review	Sedentary behavior interruption	Adults at risk for type 2 diabetes	Core	Mostly acute postprandial outcomes; at-risk subgroup.
[24]	Chueh, 2022	Systematic review	Sedentary behavior interruption	Adults	Core	Acute cognitive outcomes; short duration.
[25]	Shakerkavar, 2025	Systematic review	Movement breaks	Office workers; neck pain focus	Secondary	Narrow outcome (neck pain); office subgroup; secondary source.
[26]	Radwan, 2022	Systematic review	Movement breaks	Office workers	Core	Small office worker studies; short follow-up.
[27]	Chastin, 2015	Meta-analysis	Sedentary behavior interruption	Adults; mixed populations	Core	Early meta-analysis; observational/acute markers; residual confounding.
[28]	Zabatiero, 2019	Systematic review and meta-analysis	Sedentary behavior interruption	Adults with overweight/obesity	Secondary	Overweight/obesity subgroup; heterogeneity; secondary source.
[29]	Peddie, 2013	Randomized crossover trial (RCT)	Sedentary substitution	Healthy adults	Core	Single acute crossover in healthy adults; postprandial outcome only.
[30]	Buckingham, 2019	Systematic review	mHealth/app-guided workplace activity	Workplace adults	Core	Modest, heterogeneous effects; engagement decay over time.
[31]	Dehghan Ghahfarokhi, 2022	Systematic review and meta-analysis of RCTs	Wearable- and smartphone-supported activity	Adults with overweight/obesity	Core	Overweight/obesity subgroup; short-term; device-dependent.
[32]	Rouyard, 2025	Umbrella review	Workplace intervention	Workplace adults	Core	Umbrella review; heterogeneous workplace designs and outcomes.
[33]	Reyes Sánchez, 2025	Systematic review	Low-volume HIIT	Adults with insulin resistance	Core	Insulin-resistant subgroup; small trials.
[34]	Sabag, 2021	Review	Low-volume HIIT	Adults; cardiometabolic health	Core	Narrative review; no pooled estimate.
[35]	Sultana, 2019	Systematic review and meta-analysis	Low-volume HIIT	Adults	Core	Modest effects; heterogeneous HIIT protocols.
[36]	Yin, 2023	Meta-analysis	Low-volume HIIT	Adults	Core	Protocol heterogeneity; short duration.
[37]	Reljic, 2018	Pilot intervention study	Low-volume HIIT	Sedentary adults; community setting	Secondary	Pilot; small sample; single site; secondary source.
[38]	Wen, 2019	Systematic review and meta-analysis	HIIT protocols/time-efficient exercise	Adults	Core	Protocol heterogeneity; short-term outcomes.
[39]	Abdin, 2018	Systematic review	Workplace exercise	Office-based workers	Core	Well-being outcomes; heterogeneous workplace interventions.
[40]	Iversen, 2021	Narrative review	Minimal-dose resistance training	Adults; strength and hypertrophy	Secondary	Narrative review; expert synthesis; secondary source.
[41]	Ralston, 2017	Meta-analysis	Minimal-dose resistance training	Adults	Core	Dose-response for strength; variability by training status.
[42]	Krieger, 2010	Meta-analysis	Minimal-dose resistance training	Adults	Core	Older meta-analysis; hypertrophy volume-response focus.
[43]	Westcott, 2012	Narrative review	Minimal-dose resistance training	Adults; general health	Secondary	Narrative review; general-health framing; secondary source.
[44]	McLeod, 2019	Narrative review	Minimal-dose resistance training	Adults; aging and chronic disease	Secondary	Narrative review; aging focus; secondary source.
[45]	Schoenfeld, 2017	Systematic review & meta-analysis	Minimal-dose resistance training	Adults	Core	Load comparison; not minimal-dose adherence per se.
[46]	Schoenfeld, 2019	Randomized controlled trial (RCT)	Minimal-dose resistance training	Trained men	Secondary	Single RCT in trained men; volume (not time) focus; secondary source.

**Table 2:** Strength and maturity of evidence across lifestyle-congruent model families, with key limitations and translational cautions.

Model Family	Predominant Evidence Type	Evidence Strength/Maturity	Key Inconsistencies and Limitations	Translational Caution
Low-volume HIIT	Meta-analyses and systematic reviews of RCTs	Established (strongest in this set)	Heterogeneous protocols; mostly short duration; variable affect/tolerability; free-living adherence uncertain	Efficacious for fitness and cardiometabolic markers, but requires exertion tolerance and safety screening
Sedentary substitution/movement breaks	Meta-analyses and RCTs (largely acute)	Moderate	Predominantly acute/postprandial outcomes; small samples; few hard endpoints or long-term data	Useful to interrupt sitting; sustained chronic benefit less certain
Minimal-dose resistance training	Meta-analyses (dose-response)	Moderate	Volume-response varies by training status; few real-world minimal-dose adherence trials	Strength gains achievable at low volume; hypertrophy needs more volume
Exercise snacks/PA snacks	Small RCTs plus scoping/systematic reviews	Emerging	Small, short pilots; heterogeneous protocols; limited long-term adherence and hard-outcome data	Promising for feasibility; efficacy on clinical outcomes not yet established
VILPA	Device-based cohort plus qualitative	Preliminary/associative	Associative only and UK Biobank-dominated; confounding and reverse causation; no RCTs	Do not infer causal equivalence to structured exercise; frame as opportunistic
Delivery-mediated (mHealth, wearables, workplace)	Systematic reviews, meta-analyses and an umbrella review	Modest and heterogeneous	Small effect sizes; high heterogeneity; engagement decay; access inequities	Effects modest; long-term engagement and equity unresolved

Tiering is a qualitative appraisal of the sources in Table 1, not a formal certainty grade (e.g., GRADE), which was outside the scope of this narrative review. It is intended to prevent models with markedly different support from being presented as equivalent.

**Table 3:** Proposed exercise-friction score: measurable dimensions for operationalizing exercise friction as a design tool.

Friction Dimension	What It Captures (0 = Low Friction … 2 = High Friction)	Score (0–2)
Temporal/planning burden	0 = fits spare moments, no planning; 1 = some scheduling; 2 = requires a protected planned block	0/1/2
Transition cost	0 = no change of clothes or location; 1 = minor transition; 2 = travel, changing, showering required	0/1/2
Environmental access	0 = doable anywhere; 1 = needs stairs, safe route or space; 2 = needs facility or specific environment	0/1/2
Cognitive demand	0 = automatic or simple; 1 = some attention; 2 = high decision or skill load	0/1/2
Equipment requirement	0 = none or bodyweight; 1 = minimal (bands, wearable); 2 = machines or gym equipment	0/1/2
Supervision/safety need	0 = safe unsupervised; 1 = basic guidance; 2 = screening or supervision advisable	0/1/2
Role compatibility	0 = compatible with work and caregiving; 1 = occasional conflict; 2 = frequent conflict with roles	0/1/2
Digital access/literacy	0 = no digital dependence; 1 = simple app or wearable; 2 = requires devices, connectivity and literacy	0/1/2
Total exercise-friction load	Sum of dimension scores; higher totals favor lower-architecture models and staged progression	0–16

The score is a provisional, author-derived instrument offered to make exercise friction measurable and comparable; the dimensions, anchors, and weighting require psychometric validation (construct and predictive) before clinical use. It is intended to identify the dominant friction to be targeted, not to certify a model as sufficient.

**Table 4:** Matching exercise architecture to dominant lifestyle-related friction across adult and context profiles.

Adult/Context Profile	Dominant Lifestyle-Related Friction	Candidate Lifestyle-Congruent Model *	Practical Example	Key Caution	Progression Pathway
Office worker sitting most of the day	Sedentary accumulation	Movement breaks; workplace exercise [22,23,24,25,26,27,28,32,39]	2–3 min walking, stair, or bodyweight breaks every 30–60 min	Should complement, not replace, weekly aerobic and strengthening targets	Progress from brief breaks to accumulated daily activity and weekly structured exercise.
Time-constrained but otherwise healthy adult	Lack of uninterrupted time	Exercise snacks; low-volume HIIT [10,17,18,19,33,34,35,36,37,38]	3 × 2–5 min stair-climbing or bodyweight bouts/day; 10–15 min low-volume HIIT	Progress intensity gradually; monitor perceived exertion	Progress by increasing weekly frequency, intensity control or adding resistance training.
Physically inactive adult who rejects formal exercise	Low exercise identity; low planning capacity	VILPA; routine-embedded activity [20,21]	Brisk stair climbing, uphill walking, carrying loads, fast walking during errands	Needs clear examples, graded progression, and safety guidance	Progress from incidental vigorous bouts to planned low-friction sessions if confidence improves.
Adult with caregiving or domestic responsibilities	Logistical and transition costs	Home-based micro-sessions; distributed resistance training [41,42,45,46]	5–10 min bodyweight or resistance-band routines at home	Risk of underdosing if frequency/intensity are not progressed	Progress by increasing bout frequency, resistance load or planned weekly movement targets.
Digitally engaged adult	Need for prompts, feedback or self-monitoring	Wearable-guided or app-guided distributed activity [12,13,31,32]	Wearable prompts for steps, stairs, intensity minutes, or sitting breaks	Avoid notification fatigue and excessive monitoring burden	Progress from prompts and monitoring to autonomous routines with fewer external cues.
Hybrid or asynchronous worker	Unstable routines	Flexible/autoregulated exercise [10,16,17,18,19,20]	Weekly movement targets achieved through variable short bouts or brief sessions	Requires simple self-regulation rules	Progress from flexible weekly targets to more stable movement anchors.
Older adult or low-confidence adult	Safety concerns; low confidence; low access	Low-intensity activity snacks; supervised progression; home-based exercise [10,17,18,20,21]	Sit-to-stand bouts, short walks, light stair practice, balance-integrated breaks	Requires fall-risk consideration, functional adaptation and progression matched to mobility and confidence	Progress gradually toward longer walks, strength, balance and supervised activity when needed.
Adult with cardiometabolic risk	Need for efficient but safe stimulus	Low-volume HIIT; walking intervals; supervised progression [33,34,35,36,37,38,40,41,42,45,46]	Short interval walking or cycling sessions with gradual progression	Requires screening for contraindications, graded intensity progression, symptom monitoring, and adverse-event reporting	Progress under appropriate screening from low-intensity intervals to structured aerobic and resistance exercise.

* Equity and access note: Several of the models above assume access to safe stairs or walking environments, flexible work schedules, personal smartphones or wearables, home exercise space, and occupational autonomy. Adults in precarious employment, low-income settings, or those with functional limitations may face barriers across multiple model types. Practitioners should assess structural access alongside individual friction profiles when applying this matrix. Equity should be treated as a core design parameter of the framework rather than a peripheral caveat. Concretely, several models presuppose resources that are unevenly distributed: a personal smartphone or wearable and adequate digital literacy (digital delivery), a safe neighborhood or accessible stairs and walkable routes (VILPA, stair snacks, walking bouts), autonomy over work breaks and schedule (workplace and distributed models), and adequate home space and time free from caregiving (home-based micro-sessions). Adults in precarious or shift employment, low-income or unsafe environments, and those with disability or functional limitation may be excluded from precisely the models marketed as “low-friction.” Applying the matrix therefore requires an explicit structural-access check, and public health translation should prioritize models whose access requirements match the target population rather than defaulting to digitally or environmentally demanding options.

**Table 5:** Practical implementation pathway for lifestyle-congruent exercise: components, considerations, and responsible settings and roles.

Implementation Component	Key Considerations	Responsible Professional(S)/Setting
Delivery setting	Match setting to population and access: primary care and community centers for inactive or higher-risk adults; workplaces for desk-based workers; physiotherapy or clinical exercise services for functional or cardiometabolic limitations; digital or home-based delivery for autonomous, digitally enabled adults	Primary care, municipal/community fitness programs, physiotherapy and clinical exercise services, workplace health, digital/home
Screening and risk stratification	Baseline readiness and risk (e.g., PAR-Q+); cardiometabolic, musculoskeletal and fall-risk screening before higher-intensity (HIIT) or unsupervised vigorous (VILPA) formats	General practitioner, exercise physiologist, physiotherapist
Participant education	Explicit examples, intensity self-monitoring (talk test, RPE), safe technique, and clear rules for progression and symptom recognition	Exercise professional, physiotherapist, trained health coach
Progression model	Staged progression from low-friction entry points toward guideline-consistent aerobic and strengthening targets; adjust dose, frequency, and intensity to tolerance and confidence	Exercise physiologist or physiotherapist; self-managed with periodic review
Adverse-event monitoring	Track exertional symptoms, musculoskeletal complaints, falls, and dropouts; predefine stop or modify criteria, especially for HIIT and VILPA in higher-risk adults	Supervising professional; self-report with review pathway
Referral criteria	Escalate to medical or clinical exercise assessment on red-flag symptoms, cardiometabolic risk, functional limitation, or repeated intolerance	Primary care as referral hub to physiotherapy or clinical exercise
Outcome evaluation	Evaluate reach, adoption, adherence, maintenance, and equity (e.g., RE-AIM) alongside physiological and behavioral-fit outcomes, not physiological change alone	Program coordinator, researcher, public health team

Application in special populations. Although the framework targets general adults, its safe application differs by population and should be stated explicitly. In adults with obesity, weight-bearing formats (stairs, jumping) may need substitution with low-impact alternatives (walking intervals, cycling, seated, or supported strength), and low-volume HIIT should be introduced gradually. In type 2 diabetes and cardiometabolic risk, sedentary-substitution and short interval formats can aid glycemic control, but pre-participation screening, symptom monitoring and, where relevant, coordination with glucose-lowering therapy are required. In older adults and those with functional limitation, balance and strength integration, fall-risk assessment and supervised progression take precedence over intensity, and formats such as sit-to-stand bouts and the LiFE approach are preferable to unsupervised vigorous VILPA. Across these groups, the exercise-friction score (Table 3) should be combined with clinical screening so that the lowest-friction safe entry point is selected and progressed under supervision.

## Data Availability

No new data were created or analyzed in this study. Data sharing is not applicable to this article.

## Abbreviations

The following abbreviations are used in this manuscript:

COM-B	Capability, Opportunity, Motivation–Behavior
eHealth	Electronic health
HIIT	High-intensity interval training
LiFE	Lifestyle-integrated Functional Exercise
mHealth	Mobile health
PAR-Q+	Physical Activity Readiness Questionnaire for Everyone
RCT	Randomized controlled trial
RE-AIM	Reach, Effectiveness, Adoption, Implementation, Maintenance
UK	United Kingdom
VILPA	Vigorous intermittent lifestyle physical activity
VO_2_max	Maximal oxygen uptake
WHO	World Health Organization
